# Supplementary figures and images for: A method to quantify autonomic nervous system function in healthy, able-bodied individuals
Source: Bioelectron Med. 2021 Aug 27;7:13. doi: 10.1186/s42234-021-00075-7 (PMC8394599; doi:10.1186/s42234-021-00075-7)

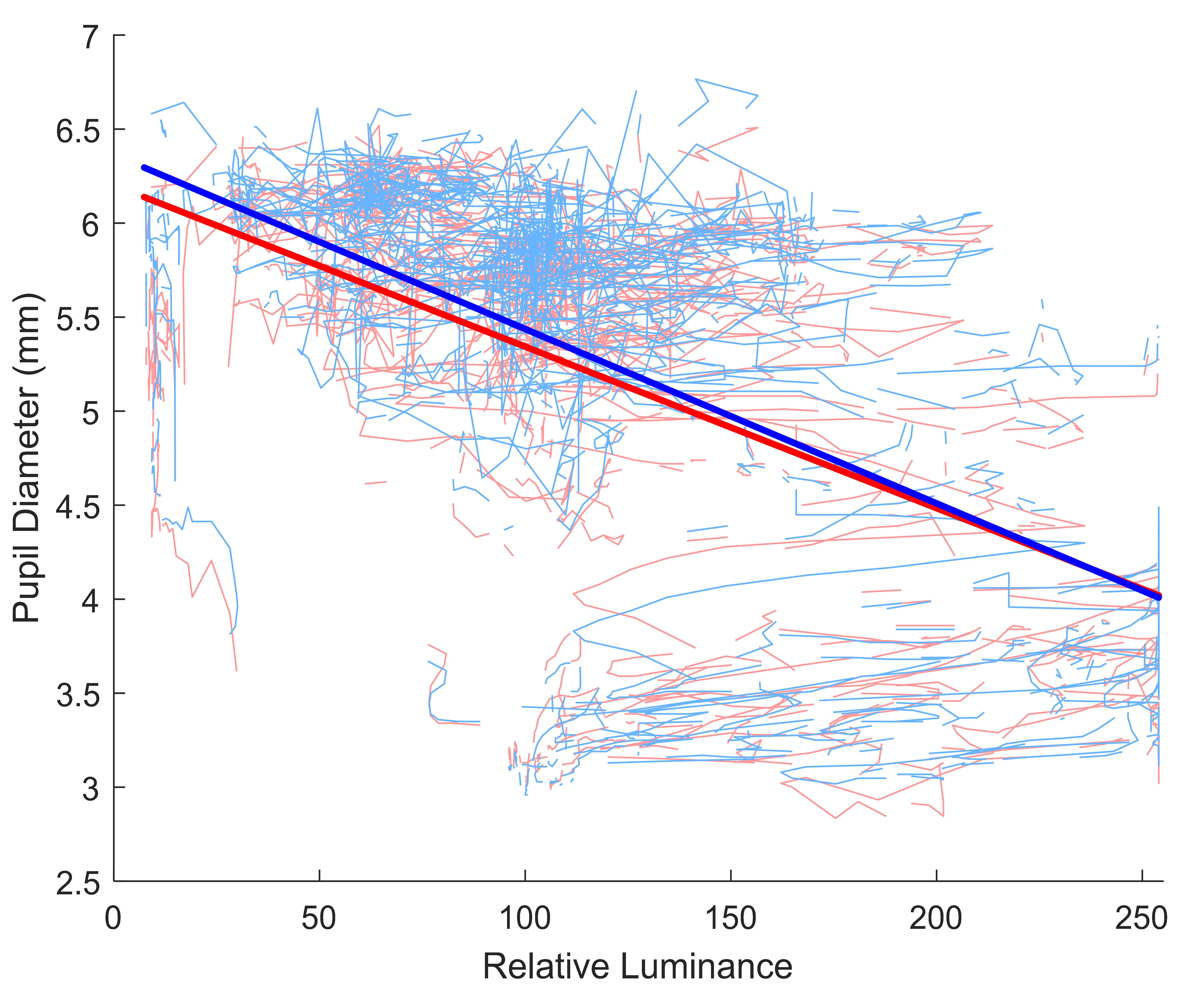

Supplement: Supplementary file 1 — Additional file 1 Supplementary Fig. 1. Normalizing pupillometry data. Gaze location was used to calibrate the pupil diameter data. The RGB values of the fixation point in each frame were converted to relative luminance (L = 0.2126R + 0.7152G + 0.0722B, based on the luminosity function) and then linearly fit to raw pupil diameter measurements. By dividing by the slope of the line, the effects of brightness were minimized. Additionally, all sessions were completed in a quiet room with lowered ambient light. [file 42234_2021_75_MOESM1_ESM.png]

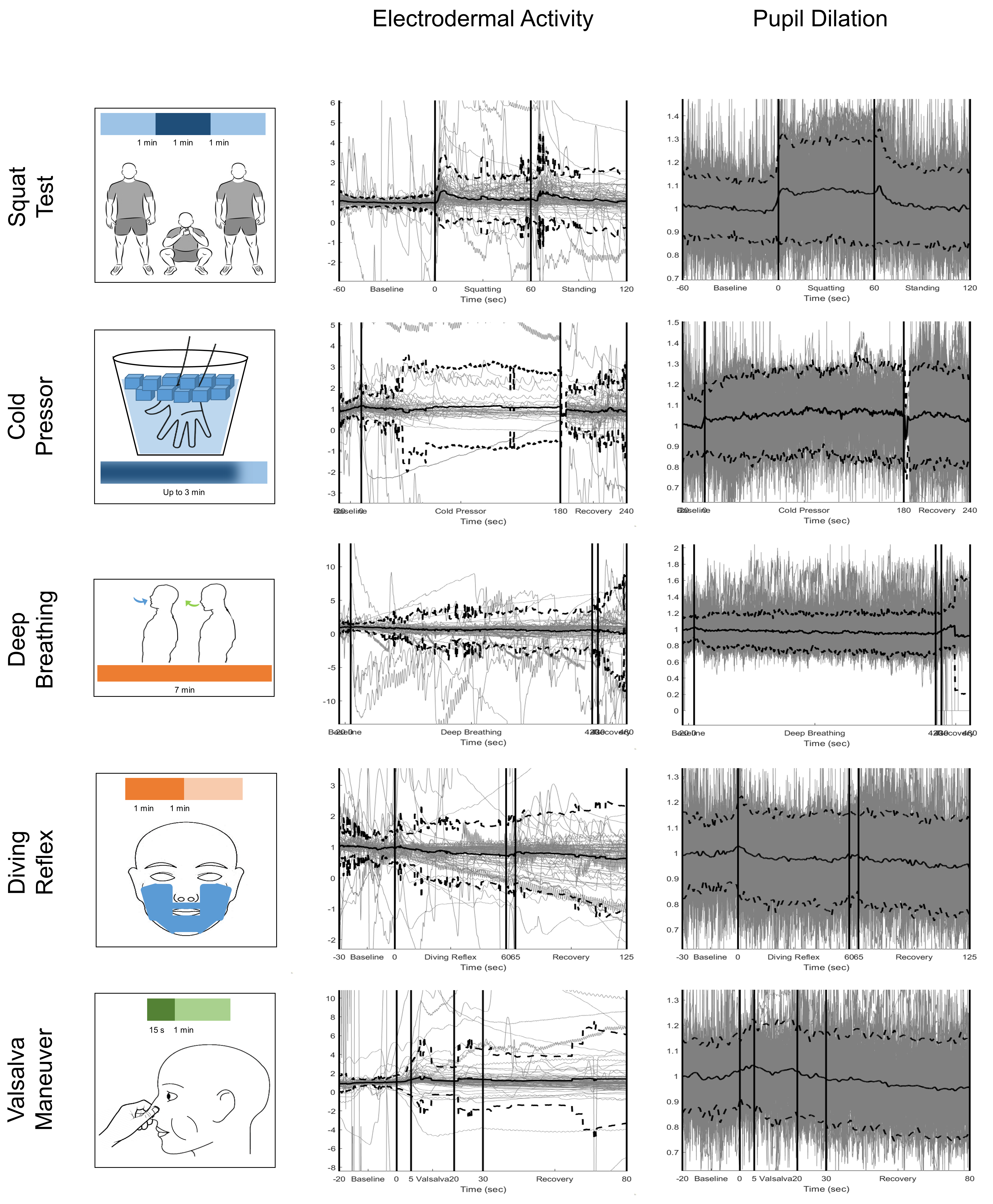

Supplement: Supplementary file 2 — Additional file 2 Supplementary Fig. 2. Average electrodermal activity and pupil dilation during autonomic testing (76 sessions). The individual calculated responses (gray lines) were accumulated and averaged (black line) to extract an average response for each modality during each test. Each column represents a different calculated signal (electrodermal activity and pupil dilation). The dotted black traces correspond to a 95% confidence interval. RMSSD was not calculated for the cold pressor test and Valsalva maneuver due to time constraints necessary to accurately convey heart rate variability. Squat Test: vertical lines reflect changes in posture from standing to squatting and then squatting to standing. Cold Pressor Test: the first vertical line reflects when the participant immersed their hand into the ice water. The second vertical line represents the maximum of three minutes. The average trace only represents the individual traces available at that time point, as participants removed their hand at their own discretion. All participants kept their hand in the ice water for at least 30 s. Deep Breathing: vertical lines reflect when the deep breathing rate (6 breaths/minute) began and ended. In the third column for the RMSSD, only the first two minutes were analyzed. Diving Reflex: vertical lines reflect when the refrigerated gel-mask was placed on and removed from the participant’s face. A five second removal period is designated before the one minute of recovery. Valsalva Maneuver: vertical lines reflect the phases of the effort, from baseline, five seconds designated for inhalation and preparation, 15 s of the Valsalva maneuver, 10 s at the end of maneuver, and a final minute of recovery. [file 42234_2021_75_MOESM2_ESM.png]
